# Lethal Prostate Cancer in Mexico: Data from the Can.Prost Mexican Registry and a Project for Early Detection

**DOI:** 10.3390/cancers16213675

**Published:** 2024-10-30

**Authors:** Miguel Angel Jimenez Rios, Anna Scavuzzo, Nancy Reynoso Noverón, Caleb García Arango, Ivan Calvo Vazquez, Alonso Hurtado Vázquez, Oscar Gerardo Arrieta Rodriguez, Miguel Angel Jimenez Davila, Maria Chiara Sighinolfi, Bernardo Rocco

**Affiliations:** 1 Department of Urology, Instituto Nacional de Cancerología, UNAM—Univesidad Nacional Autónoma de Mexico, Mexico City 14080, Mexico; incanurologia@gmail.com (M.A.J.R.); migueloajd@gmail.com (M.A.J.D.); 2 Department of Epidemiology, Instituto Nacional de Cancerología, Mexico City 14080, Mexico; reynonove1@gmail.com (N.R.N.); caleb.garc@gmail.com (C.G.A.); 3 Opus Campaign, Department of Urology, Instituto Nacional de Cancerología, Mexico City 14080, Mexico; 4 Instituto Nacional de Cancerología, Mexico City 14080, Mexico; 5 ASST Santi Paolo and Carlo, 20147 Milan, Italy; sighinolfic@yahoo.com; 6 Fondazione Policlinico Universitario Agostino Gemelli IRCCS, Università Cattolica Sacro Cuore, 00168 Rome, Italy; bernardo.rocco@gmail.com

**Keywords:** prostate cancer, aggressive features, lethal cancer, early diagnosis

## Abstract

Introduction: Epidemiological data are crucial for adopting primary and secondary prevention strategies and to develop screening protocols against prostate cancer (PCa). Despite the comprehensive characterization of PCa across White and Black men, there is a lack of data from the Mexican population. This manuscript presents data from the Can.Prost registry that captures PCa trends over the past two decades in Mexico City; furthermore, we aimed to compare clinical differences and oncological outcomes before and after the promotion of early detection actions through a campaign against PCa that occurred in 2014. Methods: A retrospective observational study on newly diagnosed Mexican PCa patients was carried out at the Instituto Nacional de Cancerología (INCan) in Mexico City. During 2014 and 2015, a project for the early diagnosis of PCa (“OPUS program”) was launched in the aforementioned tertiary hospital. Starting at the age of 45 years, all men were invited for a PSA measurement and a specialist urologist consultation. All individuals with clinical or biochemical suspicion of PCa (PSA > 4 ng/mL), in the context of age and prostate volume, underwent ultrasound-guided transrectal prostate biopsy. Then, patients with pathologically confirmed prostate cancer were stratified according to the year of diagnosis: Group A accounted for those diagnosed between 2000 and 2014 and Group B for those patients diagnosed in the timeframe of 2015–2021. Comparisons of PCa characteristics, treatment modalities and oncologic outcomes between Group A and B were performed. Results: Overall, we collected data from 2759 PCa patients from 2000 to 2021. The median PSA at baseline was 32 ng/mL, and 25% had a family history of PCa. Overall, 25.8% were asymptomatic and 46% had a non-metastatic presentation. After the OPUS campaign, PSA at diagnosis was significantly lower across all age groups. The incidence of PCa diagnosis in asymptomatic men was higher (31.4% vs. 19.9%) and a higher proportion of men were diagnosed with organ-confined, palpable disease (46% vs. 28%) (*p* < 0.001). The rate of patients eligible for active/radical treatment was higher after the OPUS campaign (patients who received surgery increased from 12.78% to 32.41%; patients who underwent radiation increased from 28.38% to 49.61%). The proportion of patients diagnosed with non-clinically significant disease was negligible and remained stable across time. Conclusions: PCa in Mexican patients displays aggressive features at diagnosis, whereas the rate of non-significant disease is negligible. The introduction of early detection strategies may lead to lower symptomatic and metastatic PCa and higher opportunities for radical treatment. This emphasizes the need for public awareness and for adjustment of screening strategies to the peculiarities of the Mexican population.

## Simple Summary

Prostate cancer (PCa) is a heterogeneous disease and the second most diagnosticated cancer in men. Epidemiological information is essential to adopt the strategy of early detection. In Mexico there is paucity of epidemiological data. We observed that Mexican men present aggressive disease at diagnosis with metastatic symptoms. Seems the Mexican race is a risk factor for PCa, as African men. “OPUS program “ is the actual screening protocol for Mexican men. We describe the concernment of early detection of PCa in the Mexican population to reduce the rate of metastases.

## 1. Introduction

Globally, in 2020, a total of 1,414,259 new cases of prostate cancer (PCa) and 375,304 related deaths were reported [1]. In the United States, according to 2023 data from the American Cancer Society, PCa—along with lung and colon cancers—is the most diagnosed cancer in men, with 288,330 new cases (29%) and an estimated 34,700 deaths in 2023. In 2019, PCa ranked 16th among the most common causes of death among men worldwide. From 2014 to 2019, the incidence of PCa increased by 3% and mortality rates remained high among men of African descent, who continue to benefit the most from early screening [2]. In 2019, an estimated 26,742 new cases and 7457 deaths due to PCa were reported in Mexico, resulting in the eleventh-highest mortality rate among Mexican men [3,4]. Despite the absence of population-based cancer registries in the country and the hospital records, there is evidence that the number of PCa cases in Mexico is underestimated and diagnosed at late stages [5,6,7,8].

Epidemiological data are crucial for adopting primary and secondary prevention strategies and to develop screening protocols against PCa. This manuscript presents data from the CanProst registry that is designed to capture PCa trends over the past two decades in a tertiary hospital in Mexico City. The registry outlines the clinical profile, treatment practice and oncological outcomes of newly diagnosed PCa patients; furthermore, we aimed to compare clinical differences and oncological outcomes before and after the promotion of early detection actions that occurred in 2014.

## 2. Materials and Methods

A retrospective observational study of newly diagnosed Mexican PCa patients was carried out at the Instituto Nacional de Cancerología (INCan) in Mexico City and was institutionally approved (protocol number 016/023/URI, CEI/1050/16). Data were collected through a digital platform and checked by an expert clinician in uro-oncology to ensure quality and consistency. Periodic and random reviews were conducted to guarantee the integrity and accuracy of data collection.

During 2014 and 2015, a project for early diagnosis of PCa (“OPUS program”) was launched in the aforementioned tertiary hospital. By starting at the age of 45–90 years were invited for a PSA measurement and a specialist urologist consultation. We attracted patients by advertising a free screening program and involving primary care physicians. All individuals with a biochemical suspicion of PCa (PSA > 4 ng/mL), in the context of age and prostate volume, or a positive digital rectal examination underwent ultrasound-guided transrectal prostate biopsy. Patients without suspicion or without family history were discharged from the program. Those with a family history or with suspicion were monitored annually with PSA and digital rectal examination to detect any potential development of PCa. For those not diagnosed with prostate cancer despite the biopsy, follow-up and a second biopsy guided by ultrasound and MRI were conducted. MRI was not used in the primary diagnosis. Local staging was performed with CT and/or MRI; metastatic staging was conducted with a CT scan, bone scan or PSMA/PET. For clinical classification, the TNM system version 2009 was used.

Patients with pathologically confirmed prostate cancer were stratified according to the year of diagnosis: Group A accounted for those diagnosed in 2000–2014 and Group B for patients diagnosed in the timeframe of 2015–2021.

The variables included were baseline demographics, the presence or absence of symptoms at diagnosis, PSA level, pathological variables (Gleason score), primary treatment (surgery vs. radiotherapy vs. androgen deprivation), presence or absence of castration-resistant PCa, and patient status at the last consultation.

### Statistical Analysis

A descriptive analysis of the whole cohort, ranging from 2000 to 2021, was undertaken. Then, comparisons between Group A and Group B were performed with regards to sociodemographic, clinical, pathological and treatment variables of PCa patients. Central tendency and dispersion measures were used for quantitative variables and frequency was used for qualitative variables. Pearson’s chi-square test, the Mann–Whitney U test or Fisher’s exact test were used, as appropriate, to evaluate differences between patients treated before and after 2014/2015 based on the TNM and PSA distribution of patients <70 years vs. >70 years. The influence of PSA level, age and Gleason score on cancer-specific survival was estimated using the log-rank test. All analyses were performed using STATA (v14.2, College Station, TX, USA) with an author’s license. A bilateral *p*-value < 0.05 was used to determine statistical significance.

## 3. Results

### 3.1. Descriptive Analysis

A total of 2759 patients diagnosed with prostate cancer (PC) were included in the study: 1349 cases from the period 2000–2014 (Group A) and 1413 cases from the period 2015–2021 (Group B). The average age at diagnosis in the analyzed sample was 67 years (SD ± 8.73); 58.13% (n = 1604) were aged less than 70 years. In terms of education level, 14.02% of the patients were illiterate, 23.63% had received a primary education, 34.46% had received a secondary education, 9.56% had completed high school, 2.24% had a technical education, and 16.05% had a university degree or higher. Regarding occupation, 55.67% of the patients were employed and 44.32% were unemployed (Table 1).

Overall, 25.73% had a familial history of PCa and PSA at baseline was 32 ng/mL.

Approximately 60.23% of patients presented urinary symptoms at the time of diagnosis, while 25.87% were asymptomatic. Of the reported PCa cases, 99% were adenocarcinomas, 0.2% (n = 6) were neuroendocrine prostate cancers, and 0.9% (n = 25) included rhabdomyosarcomas, sarcomas, and poorly differentiated carcinomas. Other clinical characteristics—including smoking status, family history and diagnostic modality—and overall pathological features are shown in Table 2. All patients received treatment, including those in ISUP group 1 (6.3%) because these cases presented pathological T2 disease.

### 3.2. Comparison Between 2000–2014 and 2015–2021

Several PCa features varied across time with the implementation of early detection initiatives. After 2015, fewer patients were diagnosed with a PSA > 100 ng/mL; Table 3 reports the PSA at diagnosis in Group A and B by age group. The average PSA level in deceased patients before the intervention was 652.4301 ng/mL (SD ± 1669.363), and after the intervention it was 405.57 ng/mL (SD ± 809.15). Only 7.5% in Group A and 9.5% in Group B presented a PSA < 4 ng/mL.

The incidence of PCa diagnosis in patients aged 60–69 years was higher in Group B compared to Group A (*p* < 0.001). The incidence of PCa diagnosis in asymptomatic men was higher in Group B (31.49%) compared to Group A (19.99%) (*p* < 0.001).

A higher proportion of men were diagnosed with organ-confined, palpable disease (46%; 651/1413) in Group B compared to Group A (28%; 378/1346) (*p* < 0.001). A higher number of patients were diagnosed with a GS 7 in Group B compared to Group A, whereas the proportion of patients diagnosed with a GS 6 remained stable (4%).

Likewise, the differences between the periods of 2000–2014 and 2015–2021, in terms of stage at diagnosis, correlate with a greater number of treatments with curative intent, such as radiotherapy or surgery, in 2015–2021.

All these differences between time periods are shown in Table 4.

The change in treatment practice is more pronounced and statistically significant in the age groups 50 to 59, 60 to 69 and 70 to 79 years. In the 50–59 age group, the proportion of patients who received surgery increased from 15.38% in 2000–2014 to 32.66% in 2015–2021 (*p* < 0.001); radiotherapy increased from 29.92% to 48.74% (*p* < 0.001) and chemotherapy increased from 9.62% to 22.11% (*p* < 0.001). In the 60 to 69 age group, surgery increased from 13.83% to 38.88% (*p* < 0.001), radiotherapy increased from 29.66% to 52.12% (*p* < 0.001) and chemotherapy increased from 9.22% to 42.64% (*p* < 0.001). Some other differences in therapeutic management by age group are shown in Table 5.

Hormone blockade use remained relatively stable in most age groups, with few significant changes. The number of surgeries before the intervention was 172, increasing to 458 afterward; a greater proportion of patients aged 60 to 69 years received surgical treatment between 2015 and 2021 (Table 5).

**Figure 1:**
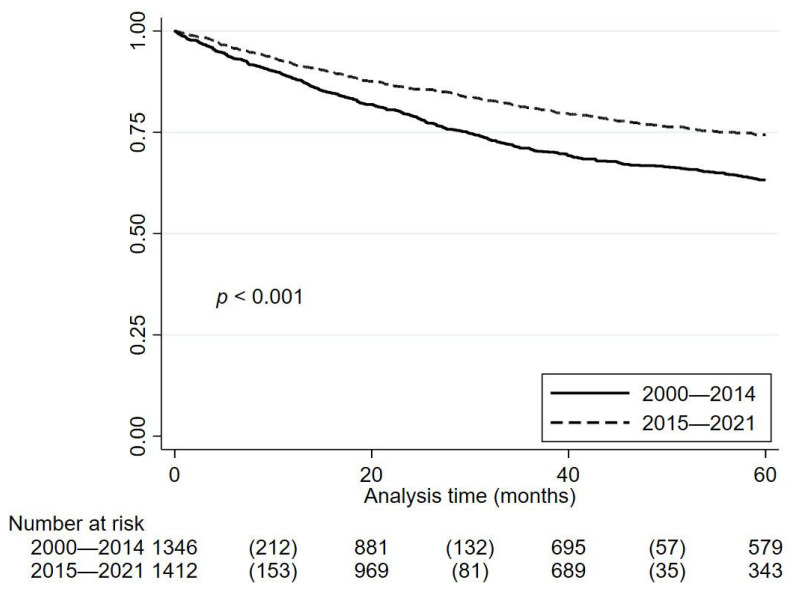
Kaplan–Meier survival estimates before and after 2014/2015.

**Figure 2:**
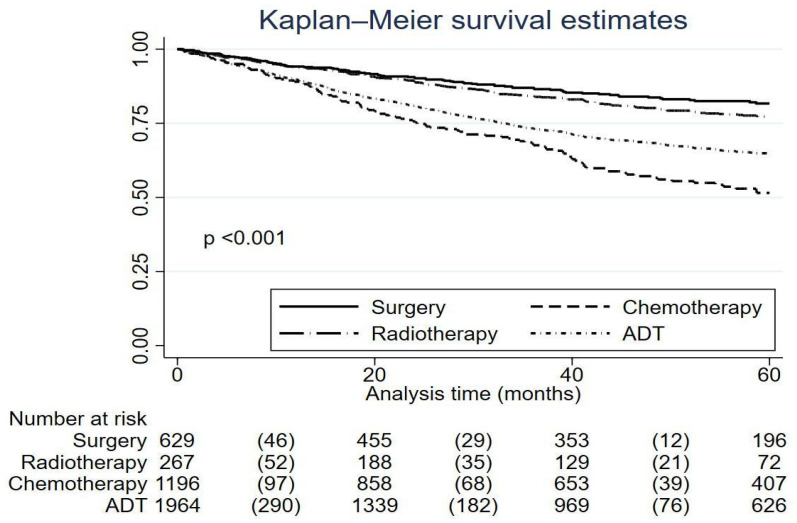
Kaplan–Meier survival estimates according to different treatments.

Despite the lower follow-up, a significant reduction in cancer mortality was observed in the second period (*p* < 0.001), with the average age of deceased patients being similar in both periods (67.2 years in 2000–2014 and 67.68 years in 2015–2021). Kaplan–Meier survival estimates are depicted in Figure 1 and Figure 2.

## 4. Discussion

In Mexico City, the introduction of an early detection strategy against PCa led to a lower rate of symptomatic PCa at diagnosis, a lower proportion of men with PCa with aggressive features and greater opportunity for radical treatment; the diagnosis of non-clinically significant PCa remained negligible across time.

Furthermore, the epidemiological data provided in this study underscores the importance of detecting and managing prostate cancer in Mexicans, a cohort in which a higher proportion of aggressive and lethal diseases are seemingly evident compared to others [5,6,7]. Understanding that possible ethnic, geographical and socioeconomic disparities may occur is crucial. In the United States, after the United States Preventive Services Task Force’s (USPSTF’s) recommendation against PSA screening for men of all ages in 2012 [8,9,10,11,12,13,14], a disproportionally greater increase in metastatic PCa was observed among Hispanic and non-Hispanic Black men. Social and economic disparities have been proposed as barriers to early diagnosis in a setting that discourages PSA screening. Health education and rural location may be considered social determinants of risk, as well. However, even after adjusting for socioeconomic factors, racial disparities in metastatic presentations remained for Hispanic and Black men; this suggests that genetics and heredity may contribute to advanced PCa presentation, too. It is recognized that Black men have a 1.76 higher chance of being diagnosed with PCa than White men; furthermore, Black men are 2.14 times more likely to die from the disease [15,16]. Differences in androgen receptor signaling, increased somatic and germline AR hypermutations, and increased AR expression could explain the issue. Similarly, germline mutations in DNA repair genes were found to be more common in Black men than in White men. This is also the case for BRCA2, where mutations are 2.8 times more frequent in Black men [17,18] Consistently, it has been recently recommended that, in Black men, screening should start by the age of 40–45 and, depending on PSA value and health status, an annual screening should be strongly considered [19].

Prostate cancer in Mexicans may behave similarly, and until now, the issue has been scarcely addressed. In 2009, an epidemiological study on PCa screening reported aggressive diseases in the Mexican population, with findings of high-grade lesions [7]. In general, diagnosis in advanced clinical stages is a challenge in Mexico, as was reported in a recent study on spinal cord metastases. Soto et al. analyzed reports from 326 patients with spinal metastases from 2010 to 2017 in three reference centers in Mexico. In males, prostate cancer was the leading cause of spinal metastases, accounting for 31.9% of cases; PCa metastasis at this site is less frequent in Asia (5%) and in North America (7%) [20].

In the current data series from Mexico City, only 25% of PCa patients are asymptomatic at diagnosis and only 46% had a non-metastatic presentation. This evidence demonstrates the need for early diagnosis actions.

The campaign promoted at the Instituto Nacional de Cancerología (INCan) in Mexico City attempted to address this healthcare issue. An improved rate of diagnosis at an asymptomatic stage and with lower PSA values occurred after the campaign; this translated into an increased rate of less aggressive and more curable disease, including a higher proportion of GS 7 and organ-confined PCa. This led to a higher proportion of men that were eligible for active—and, hopefully, radical—treatment, such as surgery or radiation (32.41% and 49.61% vs. 12.77% and 28.38%, respectively). The relatively recent introduction of agents such as docetaxel may have accounted for a higher number of patients undergoing chemotherapy in the second timeframe, too.

The Mexican experience corroborates the importance of timely PCa detection, even if based on PSA measurements and digital rectal exams. The effect of recommendation against PSA testing—such as that of the USPSTF in 2012—has led to an increase in metastatic PCa incidence at diagnosis and locally advanced cases resulting in radical prostatectomy [9,10,11,12,13,14]. On the contrary, data from the ERSPC study after 21 years of follow-up continue to indicate that early detection reduces PCa mortality [21,22,23,24,25,26,27,28].

Some considerations may arise from the current study.

Given the higher prevalence of more aggressive PCa in the Mexican population [3,4,5,6,7], screening models from European or North American countries could be less effective and reproducible in Mexico. Newly suggested screening models for PCa from the ProScreen trial [21,22] may not be applicable to the Mexican population, given that clinically insignificant cancer is observed less frequently and PCa presents with elevated PSA and Gleason levels in this population. Remarkably, the introduction of actions for early diagnosis in this study did not lead to an overdiagnosis in Mexicans, with an at least stable—and negligible—rate of unsignificant PCa diagnoses.

This is also particularly interesting considering the lack of MRI use along the diagnostic pathway suggested by the CanProst campaign: even without MRI, the proportion of GS 6 was less than 4%, suggesting a racial or genetic-driven tendency toward more aggressive disease.

The introduction of urological consultation for PSA values as low as 4 ng/mL may have mitigated the absence of MRI: proper counseling that considers age, family history and prostate volume through DRE would have balanced the risk of PCa and prompted correct indications for prostate biopsy.

The risk of overdiagnosis and overtreatment of PCa, as evident when screening is applied without an individualized risk strategy for each patient [29], may not occur in the Mexican population, given the baseline prevalence of metastatic disease. Consistently, elderly Mexicans seem to benefit from the detection and treatment of PCa, and a significant number of them could benefit from access to PCa treatment and management.

Racial differences should be considered within timely PCa detection programs [23,24]. The high rate of familial PCa may suggest that PCa in Mexican patients manifests more aggressively compared to other populations. It is likely necessary to implement timely detection programs throughout the Republic of Mexico and investigate if there are genetic differences that could justify such biological behavior.

Finally, another notable finding is that 21.5% of diagnoses occurred after transurethral resection of the prostate (TURP) or prostatic adenomectomy; in the literature, incidental PCa diagnosis following these types of treatments varies from 4 to 16% [30,31,32,33]. The incidental diagnosis of PCa following TURP negatively impacts functional and oncological outcomes when patients subsequently undergo radical prostatectomy [34,35]. Indications for benign surgery for outlet obstruction should be limited and symptoms should be investigated in view of the high prevalence of symptomatic PCa in Mexicans.

The current article is not devoid of limitations.

First, the study enrolled only patients with full data collection; the number of patients with missing data is unknown, and they may have accounted for different PCa behaviors.

Similarly, the campaign was keen to include patients from Mexico City and its metropolitan area, but data from other areas in Mexico were not collected.

Second, the lack of standardization of MRI use in the screening pathway may have resulted in less accurate local staging. Third, the widespread use of PSMA/PET in the last timeframe may have contributed to an increased incidence of metastatic diagnosis after the early detection actions in 2015.

To avoid overdiagnosis and overtreatment in the Mexican population, it could be suggested that PSMA PET/CT be used to guide prostate biopsy, as was suggested in the recent clinical trial PROSPET-BX [36].

Recognizing these limitations, we remark that this is the first and wider series de-scribing PCa characteristics among Mexican men and it argues the generalizability of screening policies based in view of possible racial, genetic and socioeconomic diversity.

## 5. Conclusions

In Mexico City, the introduction of an early detection strategy against PCa led to a lower rate of symptomatic PCa at diagnosis, a lower proportion of men with aggressive PCa features and a greater opportunity for radical treatment; the diagnosis of non-clinically significant PCa remained negligible across time. These outcomes highlight the importance of early detection programs in Mexico and introduces the need for educational programs—directed to medical and surgical community as well to improve public health awareness. This is also crucial for adjusting screening and management strategies to the peculiarity of the Mexican population.

## Figures and Tables

**Table 1:** Sociodemographic characteristics of the population.

	N = 2759	(%)
**Year of diagnosis**		
2000 to 2014	1349	48.9
2015 to 2021	1410	51.1
**Age (years)**		
41 to 49	57	2.07
50 to 59	459	16.64
60 to 69	1088	39.43
70 to 79	932	33.78
>80	223	8.08
**Educational level**		
Illiterate	387	14.02
Primary education	652	23.63
Secondary education	951	34.46
High school	264	9.56
Technical education	62	2.24
Bachelor’s degree or higher	443	16.05
**Occupation**		
Employed	1536	55.67
Unemployed	1223	44.32

**Table 2:** Clinical characteristics of the population.

	(N = 2759)	(%)
**Diagnostic clinic**		
Asymptomatic	714	25.87
Bone pain	209	7.57
Urinary symptoms	1662	60.23
Medullary section and others	174	6.3
**Smoking**		
No	1760	63.79
Yes	999	36.2
**Family history of cancer**		
No	2049	74.26
Yes	710	25.73
**Diagnostic modality**		
Prostate biopsy	1980	71.76
Biopsy elsewhere	96	3.47
Clinical PSA and/or DRE	36	1.3
TURP/adenomectomy	594	21.52
Other methods	5	0.18
**Gleason score**		
6	175	6.34
7	974	35.30
8	547	19.82
9	873	31.64
10	85	3.08
N/A	105	3.80
**Gleason pattern**		
3 + 3	175	6.34
3 + 4	425	15.4
4 + 3	549	19.89
4 + 4	514	18.62
3 + 5	20	0.72
5 + 3	13	0.47
4 + 5	692	25.08
5 + 4	181	6.56
5 + 5	85	3.08
N/A	105	3.80
**ISUP grade**		
Grade group 1 (GA ≤ 6)	175	6.34
Grade group 2 (GS 3 + 4 = 7)	425	15.4
Grade group 3 (GS 4 + 3 = 7)	549	19.9
Grade group 4 (GS 4 + 4 = 8; 3 + 5 = 8; 5 + 3 = 8)	547	19.83
Grade group 5 (GS 4 + 5 = 9; 5 + 4 = 9; 5 + 5 = 10)	958	34.72
N/A	105	3.80
**TNM staging**		
**T**		
1a	25	0.91
1b	176	6.38
1c	629	22.80
2a	256	9.28
2b	333	12.07
2c	440	15.95
3a	310	11.24
3b	130	4.71
4	242	8.77
X	218	7.90
**N**		
0	1972	71.48
1	685	24.83
X	102	3.70
**M**		
0	1290	46.76
1a	89	3.23
1b	1124	40.74
1c	139	5.04
X	117	4.24
**Prostate cancer deaths**	781	28.30

PSA: prostate-specific antigen; TURP: transurethral resection of the prostate; DRE: digital rectal exam: T: tumor; N: node; M: metastasis; ISUP: International Society of Urological Pathology.

**Table 3:** Levels of PSA by age group (years). Comparison of 2000–2014 (before the introduction of screening in 2015) to 2015–2021.

Group A (2000–2014) PSA (ng/mL)	41 to 49 Years n (%)	50 to 59 Years n (%)	60 to 69 Years n (%)	70 to 79 Years n (%)	80 to 89 Years n (%)	Total n (%)	*p*-Value
<4	1 (3.57)	16 (6.15)	42 (8.42)	30 (6.85)	12(9.92)	101 (7.5)	χ^2^: 43.53 *p* < 0.001
4–10	6 (21.43)	64(24.62)	108 (21.64)	81 (16.49)	9 (7.44)	268 (19.91)	
11–20	4 (14.29)	35 (13.46)	85 (17.03)	63 (14.38)	8 (6.61)	195 (14.48)	
20–100	8 (28.57)	53 (20.38)	133 (26.65)	119 (27.17)	34 (28.10)	347 (25.78)	
>100	9 (32.14)	92 (35.38)	131 (26.25)	145 (33.11)	58 (47.93)	435 (32.31)	
Total	28 (100)	260 (100)	499 (100)	438 (100)	114 (100)	1346 (100)	
**Group B** **(2015–2021)** **PSA (ng/mL)**							
<4	6 (20.69)	20 (10.05)	48 (8.15)	49 (9.92)	12 (11.76)	135 (9.55)	χ^2^: 30.41 *p* = 0.016
4–10	5 (17.24)	39 (19.60)	141 (23.94)	98 (19.84)	13 (12.75)	296 (20.94)	
11–20	5 (17.24)	31 (15.58)	111 (18.85)	74 (14.98)	7 (6.86)	228 (16.13)	
20–100	4 (13.79)	54 (27.14)	152 (25.81)	140 (28.34)	38 (37.25)	388 (27.45)	
>100	9 (31.03)	55 (27.64)	137 (23.26)	133 (26.92)	32 (31.37)	366 (25.9)	
Total	29 (100)	199 (100)	589 (100)	494 (100)	102 (100)	1413 (100)	

χ^2^: Pearson’s chi-square test.

**Table 4:** Clinical characteristics and parameters of prostate cancer in Mexico. Comparison of 2000–2014 (before the introduction of the screening program in 2015) to 2015–2021 (after introduction; most recent years).

	Years of Diagnosis		*p*-Value
	Group A (2000 to 2014) n = 1346 (48.79%)	Group B (2015 to 2021) n = 1413 (51.21%)	
**Age (years)**			0.001 *
41–49	28 (2.08)	29 (2.05)	
50–59	260 (20.65)	199 (14.08)	
60–69	499 (37.07)	589 (44.68)	
70–79	438 (31.20)	494 (34.96)	
>80	121 (8.99)	102 (7.22)	
**Education level**			<0.001 *
Illiterate	226 (16.79)	161 (11.39)	
Primary education	318 (23.63)	334 (23.64)	
Secondary education	485 (36.03)	466 (32.98)	
High school	109 (8.10)	155 (10.97)	
Technical education	23 (1.71)	39 (2.76)	
Bachelor’s degree or higher	185 (13.74)	258 (18.26)	
**Occupation**			<0.001 ^~^
Employed	807 (59.96)	730 (51.66)	
Unemployed	539 (40.04)	683 (48.34)	
**Diagnostic clinic**			<0.001 *
Asymptomatic	269 (19.99)	445 (31.49)	
Bone pain	129 (9.58)	80 (5.66)	
Urinary symptoms	859 (63.82)	803 (56.83)	
Medullary section	89 (6.61)	85 (6.02)	
**Family history of PCa**	320 (23.77)	390 (27.60)	0.021 ^~^
**Smoking**	548 (40.71)	451 (31.92)	<0.001 ^~^
**Surgery**	172 (12.78)	458 (32.41)	<0.001 ^~^
**Radiotherapy**	382 (28.38)	701 (49.61)	<0.001 ^~^
**Chemotherapy**	117 (8.69)	265 (18.75)	<0.001 ^~^
**Androgen deprivation therapy (ADT)**	942 (69.96)	1023 (72.40)	0.0161 ^~^
**Prostate cancer deaths**	497 (36.92)	284 (20.10)	<0.001 ^~^
**TNM staging**			
**T**			
1a	18 (1.34)	7 (0.50)	<0.001 *
1b	128 (9.51)	48 (3.40)	
1c	488 (36.26)	141 (9.98)	
2a	91 (6.76)	165 (11.68)	
2b	128 (9.51)	205 (14.51)	
2c	159 (11.81)	281 (19.89)	
3a	95 (7.06)	215 (15.22)	
3b	32 (2.38)	98 (6.94)	
4	130 (9.66)	112 (7.93)	
X	77 (5.72)	141 (9.98)	
**N**			<0.001 *
0	1080 (80.24)	892 (63.13)	
1	193 (14.34)	492 (34.82)	
X	73 (5.42)	29 (2.05)	
**M**			
0	577 (42.87)	713 (50.46)	<0.001 *
1a	20 (1.49)	69 (4.88)	
1b	663 (49.26)	461 (32.63)	
1c	33 (2.45)	106 (7.50)	
X	53 (3.94)	64 (4.53)	
**Gleason score**			
6	120 (8.91)	55 (3.89)	<0.001 *
7	399 (29.64)	575 (40.69)	
8	261 (19.39)	286 (20.24)	
9	444 (32.98)	429 (30.36)	
10	55 (4.08)	30 (2.12)	
Unknowable	67 (4.97)	38 (2.68)	
**PSA category (ng/mL)**			
<4	101(7.50)	135 (9.55)	0.003 *
4–10	268 (19.91)	296 (20.94)	
11–20	195 (14.48)	228 (16.13)	
20–100	347 (25.78)	388 (27.45)	
>100	435 (32.31)	366 (25.90)	
	**Median (IQR)**	**Median (IQR)**	
**Age (years)**	67 (61–74)	68 (62–74)	0.152 ^+^
**Gleason score**	8 (7–9)	8 (7–9)	0.346 ^+^
**PSA (ng/mL)**	32.45 (10.5–208)	24.34 (9.71–108)	<0.001 ^+^

* Pearson’s chi-square statistical test; ^~^ proportions hypothesis test; ^+^ Mann–Whitney U test. PSA: prostate-specific antigen; T: tumor; N: node; M: metastasis; IQR: interquartile range.

**Table 5:** Prostate cancer treatments by age group (years). Comparison of 2000–2014 (before the introduction of screening in 2015) to 2015–2021.

Age Group	Treatment	2000–2014	2015–2021	*p*-Value *
**41 to 49 years**		**n (%)**	**n (%)**	
	Surgery	3 (10.71)	8 (27.59)	0.106
	Radiotherapy	9 (32.14)	15 (51.72)	0.134
	Chemotherapy	3 (10.71)	7 (24.14)	0.182
	Hormonal blockade	22 (78.57)	18 (62.07)	0.173
**50 to 59 years**				
	Surgery	40 (15.38)	65 (32.66)	<0.001
	Radiotherapy	70 (29.92)	97 (48.74)	<0.001
	Chemotherapy	25 (9.62)	44 (22.11)	<0.001
	Hormonal blockade	176 (67.69)	142 (71.36)	0.399
**60 to 69 years**				
	Surgery	69 (13.83)	229 (38.88)	<0.001
	Radiotherapy	148 (29.66)	307 (52.12)	<0.001
	Chemotherapy	46 (9.22)	114 (42.64)	<0.001
	Hormonal blockade	337 (67.54)	410 (69.61)	0.471
**70 to 79 years**				
	Surgery	46 (10.50)	135 (27.33)	<0.001
	Radiotherapy	129 (9.45)	243 (49.19)	<0.001
	Chemotherapy	38 (8.68)	82 (16.60)	<0.001
	Hormonal blockade	328 (74.89)	372 (75.30)	0.939
**80 to 89 years**				
	Surgery	14 (11.57)	21 (20.59)	0.065
	Radiotherapy	26 (21.49)	39 (38.24)	0.006
	Chemotherapy	5 (4.13)	18 (17.65)	<0.001
	Hormonal blockade	79 (65.29)	81 (79.41)	0.019
**Total**				
	Surgery	172 (12.77)	458 (32.41)	<0.001
	Radiotherapy	382 (28.38)	701 (49.61)	<0.001
	Chemotherapy	117 (8.69)	265 (18.75)	<0.001
	Hormonal blockade	942 (69.98)	1023 (72.39)	0.177

* Z-test for proportions.

## Data Availability

Data are contained within the article.
